# Who breaches the four-hour emergency department wait time target? A retrospective analysis of 374,000 emergency department attendances between 2008 and 2013 at a type 1 emergency department in England

**DOI:** 10.1186/s12873-017-0145-2

**Published:** 2017-11-02

**Authors:** Niklas Bobrovitz, Daniel S. Lasserson, Adam D. M. Briggs

**Affiliations:** 1 Nuffield Department of Primary Care Health Sciences, University of Oxford, Radcliffe Primary Care Building, Woodstock Road, Oxford, OX2 6GG UK; 2 Ambulatory Care, Institute of Applied Health Research, College of Medical and Dental Sciences, University of Birmingham, Edgbaston, Birmingham, B15 2TT UK; 3 Centre on Population Approaches for Non-Communicable Disease Prevention, Nuffield Department of Population Health, University of Oxford, Richard Doll Building, Old Road Campus, Oxford, OX3 7LF UK; 4 Health Care Policy and Practice, The Dartmouth Institute for Health Policy and Clinical Practice, Level 5, Williamson Translational Research Building, One, Medical Centre Drive, Lebanon, NH 03756 USA

**Keywords:** Four-hour target, Emergency department, Accident and emergency, Performance

## Abstract

**Background:**

The four-hour target is a key hospital emergency department performance indicator in England and one that drives the physical and organisational design of the ED. Some studies have identified time of presentation as a key factor affecting waiting times. Few studies have investigated other determinants of breaching the four-hour target. Therefore, our objective was to describe patterns of emergency department breaches of the four-hour wait time target and identify patients at highest risk of breaching.

**Methods:**

This was a retrospective cohort study of a large type 1 Emergency department at an NHS teaching hospital in Oxford, England. We analysed anonymised individual level patient data for 378,873 emergency department attendances, representing all attendances between April 2008 and April 2013. We examined patient characteristics and emergency department presentation circumstances associated with the highest likelihood of breaching the four-hour wait time target.

**Results:**

We used 374,459 complete cases for analysis. In total, 8.3% of all patients breached the four-hour wait time target. The main determinants of patients breaching the four-hour wait time target were hour of arrival to the ED, day of the week, patient age, ED referral source, and the types of investigations patients receive (*p* < 0.01 for all associations). Patients most likely to breach the four-hour target were older, presented at night, presented on Monday, received multiple types of investigation in the emergency department, and were not self-referred (p < 0.01 for all associations). Patients attending from October to February had a higher odds of breaching compared to those attending from March to September (OR 1.63, 95% CI 1.59 to 1.66).

**Conclusions:**

There are a number of independent patient and circumstantial factors associated with the probability of breaching the four-hour ED wait time target including patient age, ED referral source, the types of investigations patients receive, as well as the hour, day, and month of arrival to the ED. Efforts to reduce the number of breaches could explore late-evening/overnight staffing, access to diagnostic tests, rapid discharge facilities, and early assessment and input on diagnostic and management strategies from a senior practitioner.

## Background

In the second quarter of the 2016–2017 financial year, the percentage of patients at type 1 emergency departments (consultant led with 24 h resuscitation services) [1] breaching the government’s four-hour wait-time target in England was 9.55%, the second highest since 2003. [2] Since 2010, acute NHS provider trusts have found it increasingly challenging to meet government targets for the percentage of attendances being admitted, discharged, or transferred within four hours of arrival, which was set at 98% between 2005 and 2011, and at 95% from 2011 onwards.

Long ED waiting times are consistently associated with patient dissatisfaction, [3–6] overcrowding and further exacerbations of wait times leading to negative patient outcomes, including leaving without being seen, [7, 8] and adverse clinical outcomes. [9, 10] The four-hour target was first introduced in England in 2005 and subsequent studies have consistently found it to have reduced patient waiting times. Similar wait time targets have been implemented in Australia (4 h) and New Zealand (6 h) with some beneficial effects on patient outcomes. [11, 12] However, there is debate as to whether clinical outcomes in England have been positively affected. [13–19]

Despite controversy regarding its effect on clinical outcomes, the four-hour target is now a key hospital ED performance indicator in England [20] and one that drives the physical and organisational design of EDs. [21–23] However, few studies have investigated the determinants of patients breaching. [24, 25] Therefore, we analysed all emergency department attendances at a large acute NHS teaching hospital in Oxford, England between 2008 and 2013 to describe patterns of emergency department breaches and identify patients at highest risk of breaching. Predicting which patients are most likely to breach the four-hour target may be valuable to plan services appropriately, meet the needs of patients, and comply with wait time targets.

## Methods

This was a retrospective cohort study. All ED attendances at a large NHS teaching hospital in Oxford, England were prospectively recorded by ED clinicians and ED administrative staff using FirstNet and Millenium computer systems between April 2008 and April 2013. Anonymised individual level patient data for 378,873 ED attendances were retrospectively extracted, representing all attendances during the time period studied. Available data fields were patient age, time and date of attendance, length of stay in the ED, referral source (categorised into self-referral [walk-in], general practitioner, emergency services [brought in by ambulance], and other), and investigations whilst in the ED. We categorised investigations to determine the number of different types of investigations patients received as opposed to the number of total investigations (i.e. four x-rays = one type of investigation). We also classified the investigation codes into four groups based on their invasiveness and approximate time required to complete the investigation (Table 1). All variables were tabulated and reviewed to ensure the legitimacy of the data points. We conducted a complete case analysis. Patient records with years of age listed above 110 were excluded on the assumption of incorrect data entry (*n* = 11). 85 records were excluded because the time of discharge decision was incorrect (i.e., before the time of entry into the ED). 4318 records were excluded due to data missing for one or more variables. Sensitivity analysis showed no differences between the characteristics and breach probabilities for these patients and those included in the analysis. We sought an opinion from the joint research office classification group at the University of Oxford and they confirmed that this activity constituted service evaluation. Therefore, no ethics approval was necessary.

**Table 1:** Investigation codes and categories

Category	Investigation code
No investigations ordered	no investigations, other investigations^a^
Point-of-care tests	electrocardiogram**,** blood gases**,** pregnancy test, visual (refraction, orthoptic tests, and computerised visual fields), urinalysis
Laboratory tests	haematology, blood matching, biochemistry, urine chemistry, histology, clotting, immunological blood tests, cardiac enzyme, toxicology, blood culture, serology, bacteriology
Simple imaging	x-ray plain film
Complex imaging	computer assisted tomography (CT) scan**,** ultrasound**,** magnetic resonance imaging (MRI)

^a^Used by clinical teams as a default code if none of the other investigations listed in Table 1 were undertaken (descibes history taking and examination)

### Statistical analyses

Univariate and multivariate regression analyses were conducted using STATA version 11 (College Station, TX: StataCorp LP). We assessed patient characteristics and ED attendance variables as predictors of breaching the four-hour wait limit. Patient age was found to have a curvilinear relationship with breach probability and therefore we used a piecewise linear function (node at age four) to model this variable in regression analysis. Age four was selected as the node because at this age the nature of the correlation with breach probability changed from negative to positive. When analysing seasonality of breaching we classified October–February inclusive as high season given that these months have been shown in national analyses to correspond with increases in demand for hospital services. [26] Results are presented as odds ratios with 95% confidence intervals (CI).

Differences between patients spending less than four hours in the ED and those spending more than four hours were assessed using the Wilcoxon Rank Sum test for data that were not normally distributed. Chi-square tests with bonferroni corrected post-hoc comparisons were conducted on categorical data to identify differences in characteristics of breaching and non-breaching patients. Given the size of the dataset and associated power of analysis we set the level of significance at *p* < 0.01 to reduce the likelihood of type 1 errors.

## Results

Of 378,873 ED attendances, 374,459 had complete data and were analysed (98.8% of all attendances). Attendances to the ED fluctuated by year with the greatest number occurring between April 2011 and April 2012. Table 2 describes the characteristics of people attending the ED. The majority (69.6%) of attendees were under the age of 50. Most patients arrived between the hours of 8:00 and 20:00 (67.0%). Patients were primarily self-referred (43.8%) or referred by emergency medical services (31.2%) to attend the ED. Other sources of referral included general medical practitioner, dental practitioners, dental practices, community dental services, police, work, educational establishments, and local authority social services. The median waiting time for a decision to admit to hospital or discharge or transfer from the ED was 2 h and 59 min (Interquartile range: 1 h 55 m to 3 h 49 m) with 9.2% of all patients breaching the four-hour wait time target. The proportion of patients breaching the target varied significantly by year (*p* < 0.01) with the lowest percentage occurring in 2008/2009 (3.35%) and the highest percentage in 2011/2012 (15.4%). With regards to investigations completed in the ED, 39.9% of patients had “no investigations ordered” and 34.9% had the combination of “simple imaging, laboratory tests, and point of care tests”.

**Table 2:** Patient characteristics

Characteristics	Percent (*n* = 374,459)
Age at ED arrival	31 (17, 56)^a^
0–4	11.7
5–17	13.3
18–30	23.7
31–64	32.0
65–79	9.7
80+	9.1
Time of ED Arrival	
08:00 to 19:59	67.0
20:00 to 07:59	33.0
Source of Referral	
Self-referral	43.8
Other sources^b^	56.2
Number of attendances in the previous financial year	1 (1, 2)^a^
Number of investigation types	1 (0, 2)^a^
Investigation categories	
No investigations ordered^c^	39.3
Point of care only	3.4
Laboratory tests and point of care	15.2
Simple imaging, laboratory tests, point of care	34.9
Complex imaging, simple imaging, laboratory tests, point of care	7.2
Median length of time spent in ED	2 h 59 m (1 h 55 m, 3 h 49 m)^a,c^
Patients exceeding the four-hour ED wait target	9.2

*ED* emergency department

^a^Median and interquartile range

^b^Other types of referral include: emergency services, general medical practitioner, dental practitioners, dental practices, community dental services, police, work, educational establishments, and local authority social services

^c^h = hours, m = minutes

**Fig. 1:**
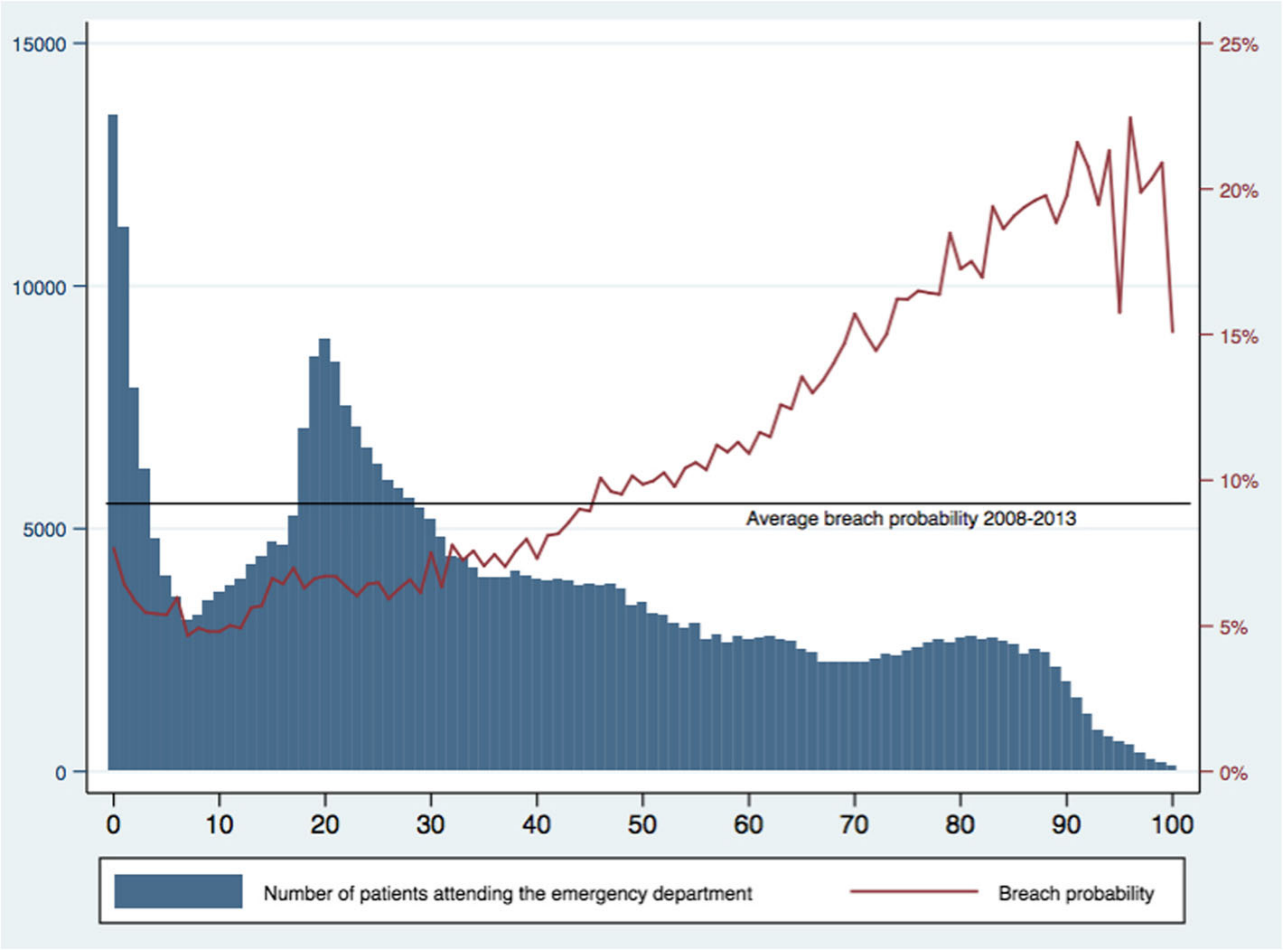
Five-year aggregated data (2008–2013) of emergency department attendances and breach probability by patient age. X-axis: Patient age in single years. Left-hand Y-axis: Number of patients attending the emergency department. Right-hand Y-axis: Probability of breaching the four hour wait target

**Fig. 2:**
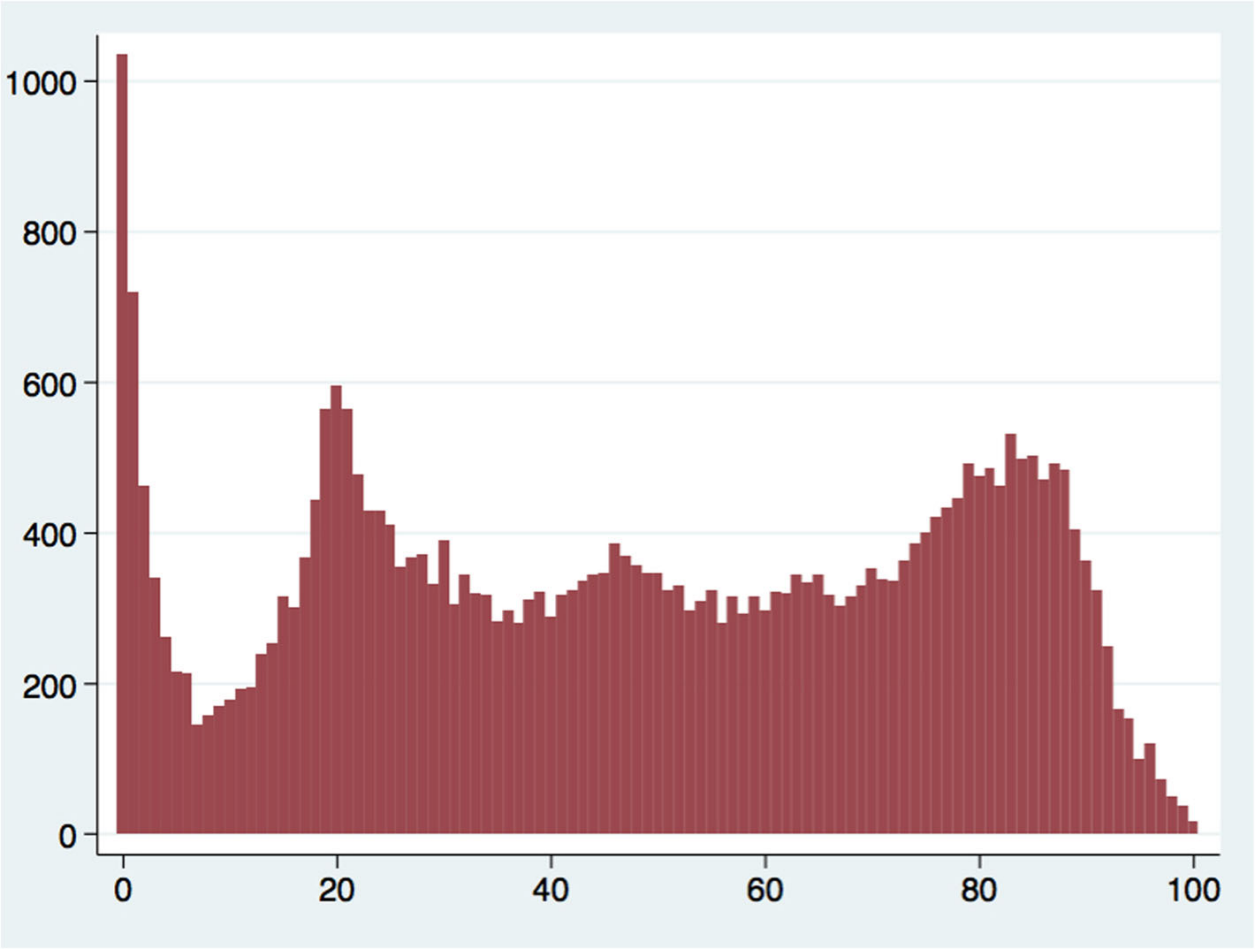
Five-year aggregated data (2008–2013) of the absolute number of emergency department breaches by patient age. X-axis: Patient age in single years. Left-hand Y-axis: Number of patients waiting more than four h in the emergency department

Figure 1 shows the age distribution of ED attendees and the probability of breaching by age. There were a large number of attendances among children ages 0–3 and younger adults ages 17–30. The largest number of breaches occurred among children less than 3 years of age, young adults ages 18–25, and among the elderly ages 75–89 (Fig. 2). Breaches in the first two groups (0–3, 18–25) appear to be driven by overall attendance numbers as the probability of breaching was low, while breaches in the third group (≥75) seem to be driven by a high breach probability, as the actual number of attendances was low in this group.

**Fig. 3:**
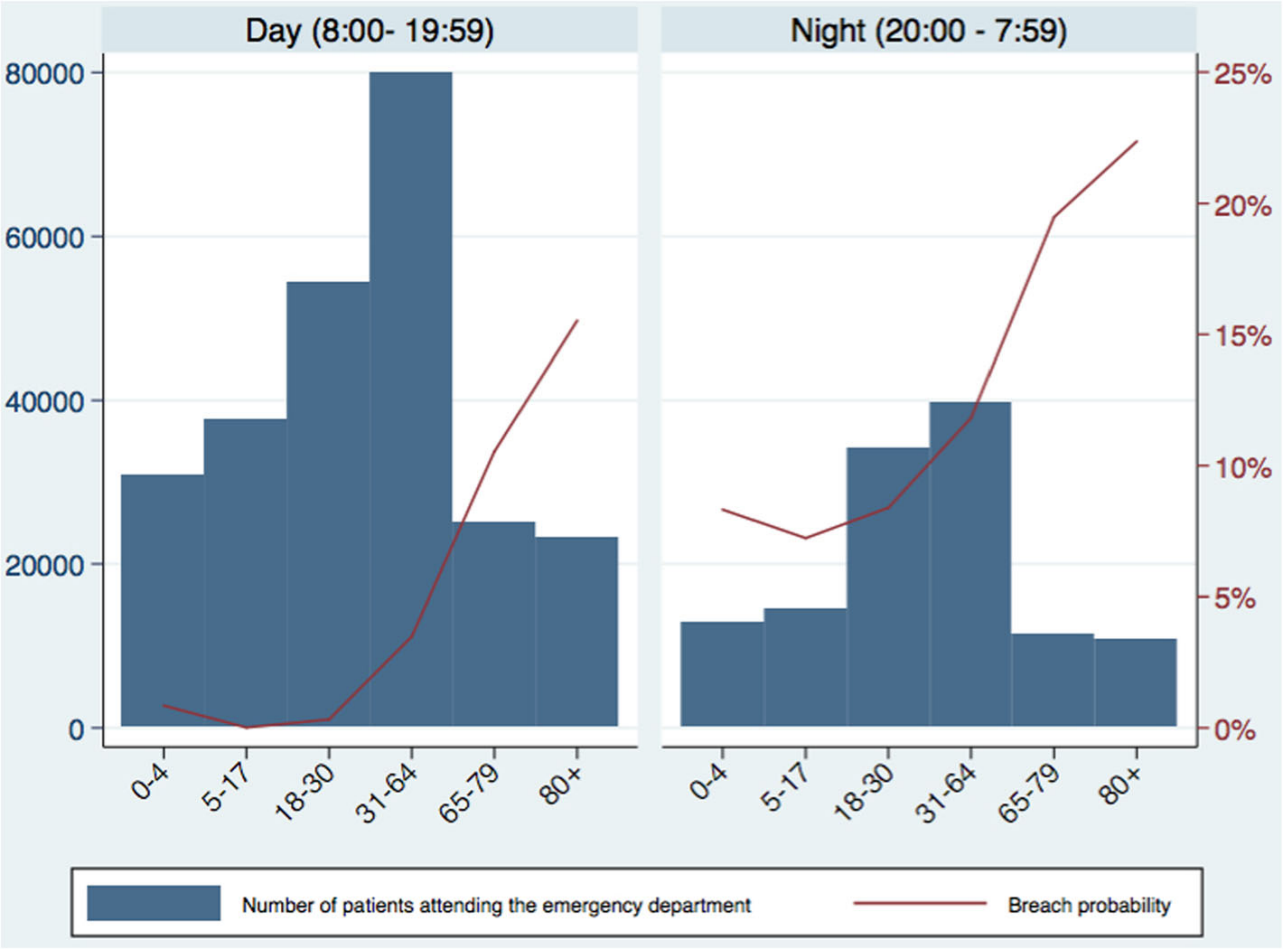
Emergency department attendances and breach probability by age categories in the day and at night. X-axis: Patient age. Left-hand Y-axis: Number of patients attending the emergency department. Right-hand Y-axis: Probability of breaching the four hour wait target

Figure 3 shows the number of patients arriving in ED and the probability of breaching during the day (8:00 to 19:59) and at night (20:00 to 7:59) by age. Although more attendances occurred in the day the probability of breaching was higher for those arriving between 20:00 and 7:59 for all ages compared to day-time attendances. The overall increase in ED breach probability for night-time attendees compared to day-time attendees was 3.6% (*p* < 0.01).

**Fig. 4:**
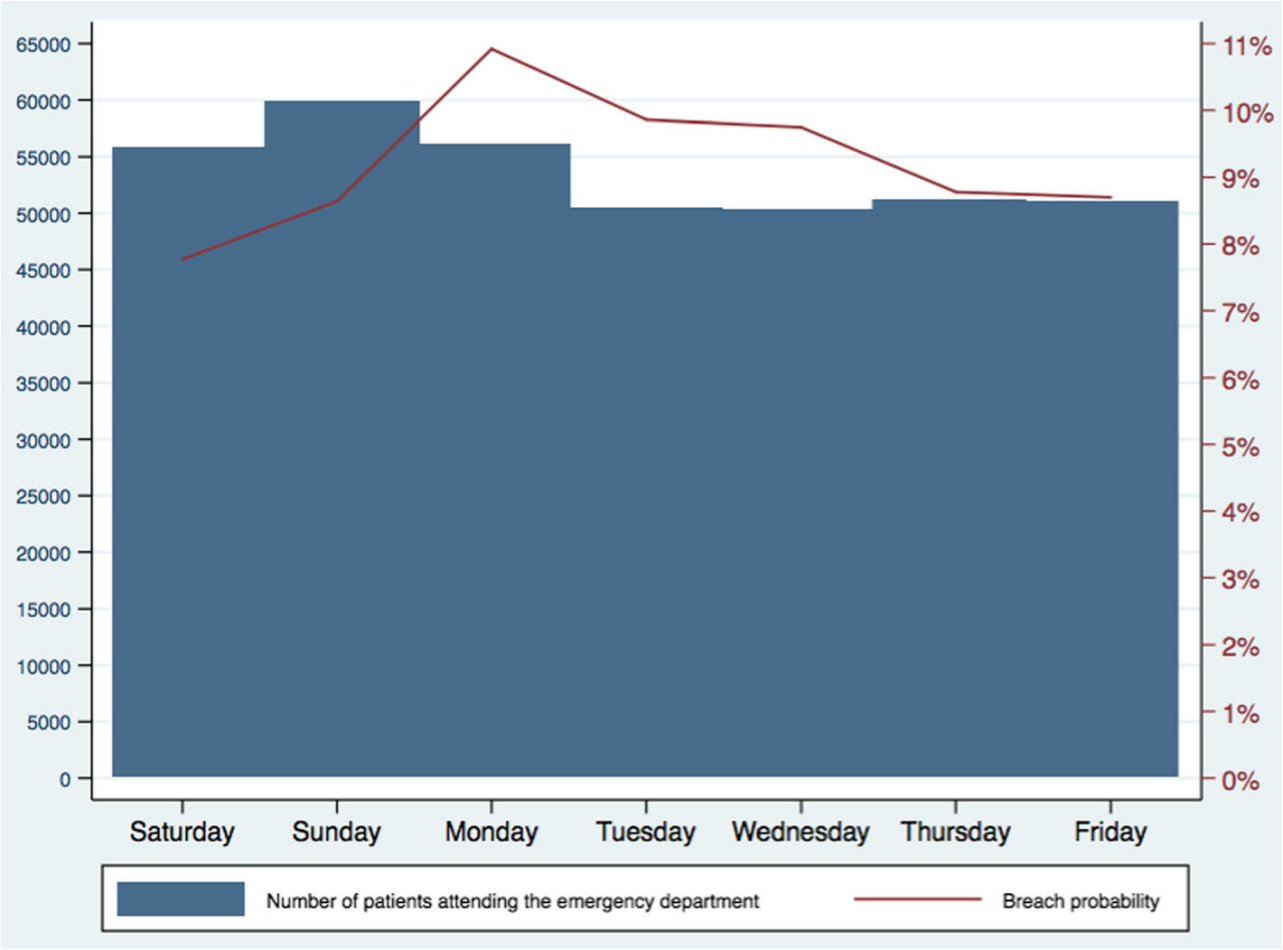
Breach probability by day of arrival to the emergency department. X-axis: Day of the week. Left-hand Y-axis: Number of patients attending the emergency department. Right-hand Y-axis: Probability of breaching the four hour wait target

The probability of breaching varied by the day patients attended the ED (Fig. 4). Although most attendances occurred on Saturday, Sunday, and Monday, the greatest probability of breaching was on Monday (10.9%), Tuesday (9.9%), and Wednesday (9.8%) while the lowest probability of breaching was on Saturday (7.8%) and Sunday (8.6%).

With regards to the source of referral to the ED, self-referred patients were less likely to breach than patients attending by any other source (5.2% vs. 12.3%, *p* < 0.001). Sensitivity analysis showed that this pattern was similar across all years (2008–2013).

**Fig. 5:**
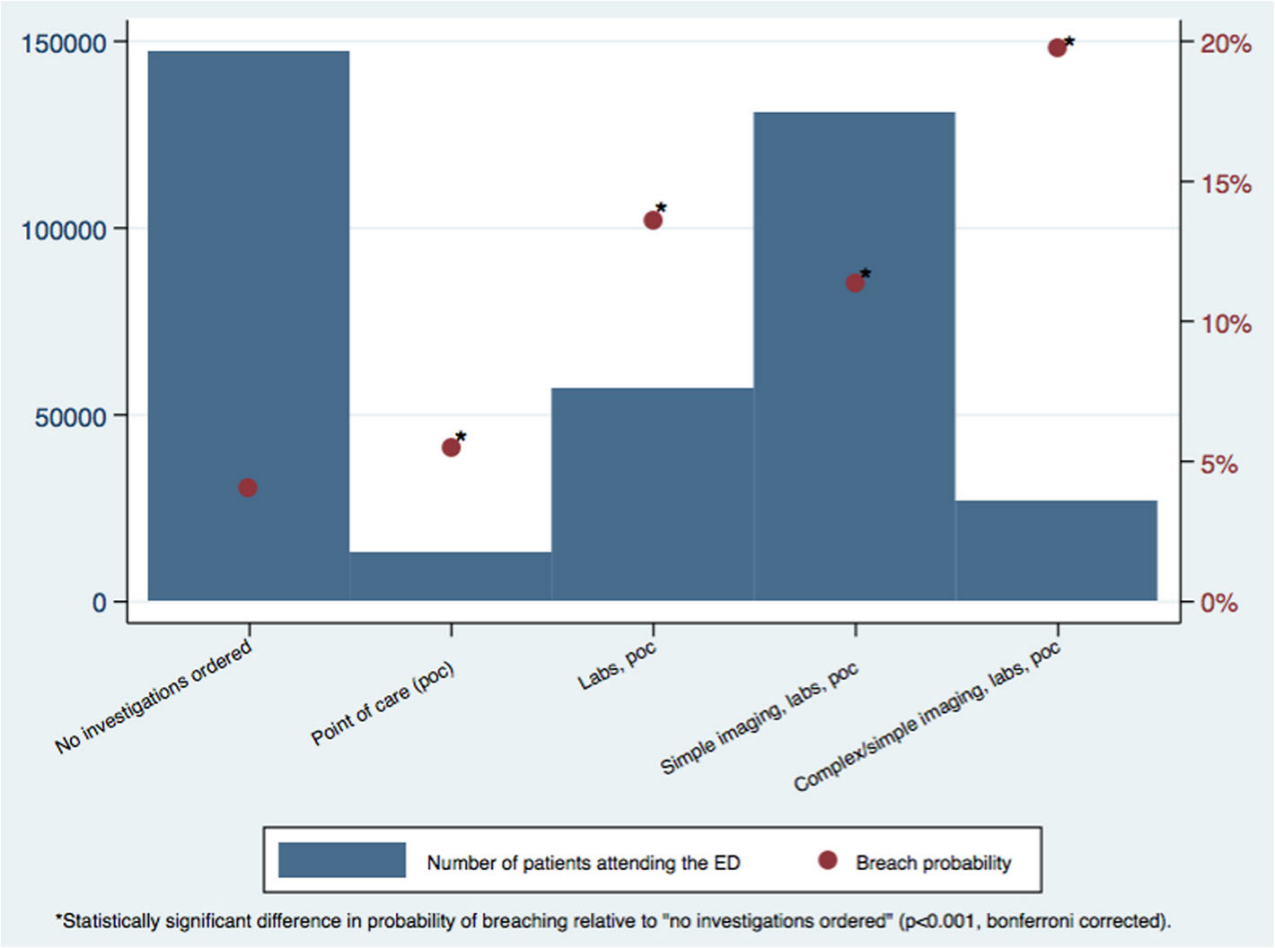
Distribution of investigation categories and the probability of breaching. X-axis: Types of investigations patients received. Left-hand Y-axis: Number of patients attending the emergency department. Right-hand Y-axis: Probability of breaching the four hour wait target

Figure 5 shows the number of patients receiving different combinations of investigations in the ED and breach probability. Most patients had no investigations ordered or received the combination of simple imaging, laboratory tests, and point-of-care tests. The breach probability was significantly higher for patients receiving investigations (*p* < 0.001) relative to patients with no investigations ordered.

The types of investigations ordered varied by the source of patient referral to the ED. Patients that were self-referred to the ED were less likely to receive multiple types of investigations compared to patients referred to the ED by other sources (p < 0.001). The types of investigations received also varied by patient age (p < 0.001). Older patients appeared to receive more types of investigations compared to younger people.

Table 3 shows adjusted odds ratios from the multivariate logistic regression. All variables were found to have a significant relationship with breaching. Strong independent predictors of waiting more than four hours in the ED included the investigations a patient received, arrival time, day of the week, referral source, and age. Patients with the greatest odds of breaching were: those receiving the most comprehensive combination of investigations (complex imaging, simple imaging, x-ray, laboratory tests, and point-of-care tests) compared to those that had no investigations ordered (OR 4.86, 95% CI 4.63 to 5.10); patients arriving during the night (20:00 to 7:59) compared to day-time (8:00 to 19:59) arrivals (OR 1.50 95% CI 1.47 to 1.54); and patients attending the emergency department on a Monday (compared with Saturday, the day with the lowest odds of breaching, OR 1.50, 95% CI 1.43 to 1.56). With regards to age, a J-shaped relationship was observed with decreasing odds of breaching up to the age of four years (with an average OR 0.84 [95% CI 0.83 to 0.85] for each additional year of age) before the odds of breaching increase with each additional year of age (1.20, 95% CI 1.19 to 1.23).

**Table 3:** Results of multivariate logistic regression to predict breaching the four h target

Variable	Odds ratio with 95% confidence interval	*P*-Value
Arrival hour^a^		
20:00–7:59	1.50 (1.47 to 1.54)	<0.001
Day of the week^b^		
Monday	1.50 (1.43 to 1.56)	<0.001
Tuesday	1.29 (1.24 to 1.35)	<0.001
Wednesday	1.27 (1.21 to 1.33)	<0.001
Thursday	1.11 (1.06 to 1.16)	<0.001
Friday	1.09 (1.04 to 1.14)	<0.001
Sunday	1.18 (1.13 to 1.23)	<0.001
Age (if younger than 4)	0.84 (0.83 to 0.85)	<0.001
Age (if 4 or older)	1.20 (1.19 to 1.23)	<0.001
Self-referral^c^	0.59 (0.57 to 0.60)	<0.001
Investigation category^d^		
Point of care	1.10 (1.01 to 1.19)	<0.001
Labs, point of care	2.41 (2.32 to 2.51)	<0.001
Simple imaging, labs, point of care	2.24 (2.16 to 2.51)	<0.001
Complex imaging, simple imaging, laboratory tests, point of care	4.86 (4.63 to 5.10)	<0.001
Number of attendances in the previous financial year	1.008 (1.006 to 1.011)	<0.001
Year^e^		
2009	1.63 (1.53 to 1.74)	<0.001
2010	2.25 (2.12 to 2.39)	<0.001
2011	4.64 (4.39 to 4.91)	<0.001
2012	4.92 (4.66 to 5.21)	<0.001
2013	4.28 (4.00 to 4.57)	<0.001
Season		
October–February inclusive (high season)^f^	1.63 (1.59 to 1.66)	<0.001

^a^Compared to arrival between 8:00–19:59

^b^The reference day was Saturday

^c^Compared to all other types of referral: emergency services, general medical practitioner, dental practitioners, dental practices, community dental services, police, work, educational establishments, and local authority social services

^d^Comparison category is “no investigations ordered”

^e^The reference year was 2008

^f^Compared to ED attendances in March–September inclusive (Low season)

Model metrics: R^2^ = 12%

**Fig. 6:**
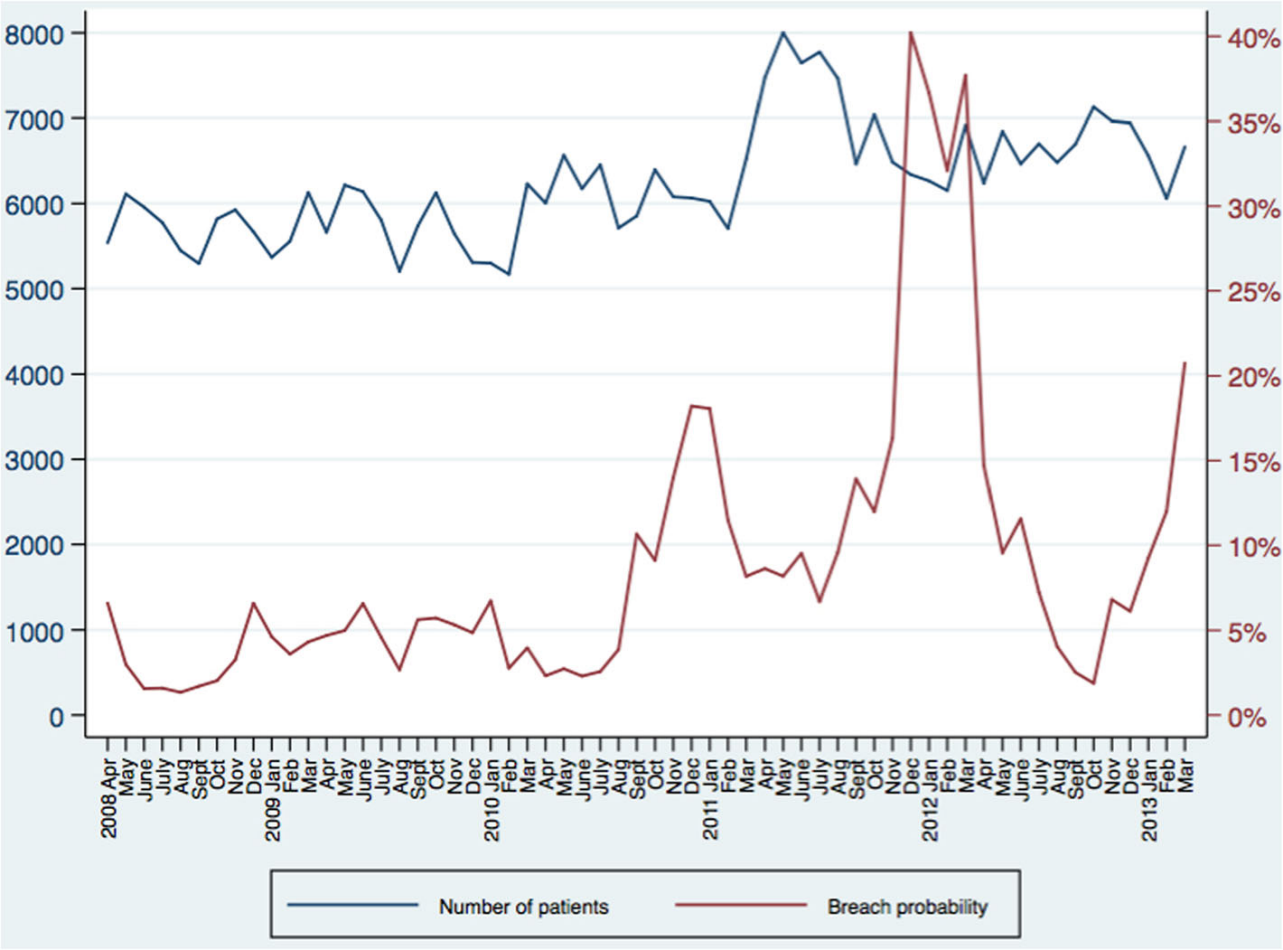
Emergency department attendances and breach probability from 2008 to 2013. X-axis: Year and month. Left-hand Y-axis: Number of patients attending the emergency department. Right-hand Y-axis: Probability of breaching the four hour wait target

Figure 6 shows the number of ED attendances and probability of breaching over time. Season of ED attendance was also significantly associated with breach probability. Patients attending from October to February had a higher odds of breaching compared to those attending from March to September (1.63, 95% CI 1.59 to 1.66). Finally, year had a very strong relationship with breaching. Relative to 2008, attendances during each subsequent year showed an increase in the odds of breaching, with a peak in 2012 (4.92, 95% CI 4.66 to 5.21).

## Discussion

We analysed over 370,000 emergency department attendances between 2008 and 2013 at a large NHS teaching hospital in Oxford, England. We found the main determinants of patients breaching the four-hour wait time target were hour of arrival to the ED, day of the week, patient age, ED referral source, and the types of investigations patients receive. We also showed a relationship with two macro-level characteristics: the year of attendance and season of attendance, which are highly likely to be markers of variability in congestion within the department.

We found that the patients most likely to breach the four-hour target were older, receiving multiple types of investigation, and not self-referred. These factors all suggest high case complexity. Delays for these patients could be due to challenges in diagnosing and choosing a care plan for complex illness including timely access to imaging and investigations, the availability of senior clinicians with expertise in medicine of older people to assess and treat patients, and the availability of supportive care in the community for patients that may not require hospital bed-based care. These explanations are also likely to explain the significantly higher probability of breaching at night.

A third finding of this study was that breach probability was highest on Mondays, Tuesdays, and Wednesdays. These were not the days of highest volume in the ED however, they did follow the weekend where ED attendance is highest. It is possible that breaches following the weekend are the result of a shortage of inpatient beds or high case complexity from patients who delay seeking care until their registered GP practice is available. [27] High bed occupancy rates, due to delays in transfers of care, have been shown to cause increases in ED wait times as physicians face difficulty admitting to in-patient beds [28]. This phenomenon has been observed in National Studies of A&E performance in England [22].

Our results are similar to those of previous authors [24, 25, 29]. For example, Goodacre et al. conducted a multivariate analysis of routine data collected on 71,000 patients in Sheffield and showed that older age, presentation at night, and presentation on Sunday and Monday were significantly associated with breach probability [24]. The authors concluded that the most important factors affecting waiting times were related to time of presentation.

Our analysis is novel as it has identified the burden of diagnostic testing as an independent predictor of breaching. We have also shown how the relationship between age and breaching differs across the age spectrum, with a large proportion of breaching cases among children and young adults being the result of high volume at a low breach probability, and a large portion of cases among the elderly due to high breach probability despite a low volume.

Our data suggest that policies to reduce the number of breaches could explore overnight staffing on select weekdays, expanding access to imaging and diagnostic tests, improving availability of senior clinicians with expertise in medicine of older people to asses and treat patients, and rapid discharge facilities [30–32]. One strategy shown in observational studies to have some success in reducing ED length of stay is early assessment and input on diagnostic and management strategy from a senior practitioner. Given the burden of diagnostic testing shown in our study, this strategy may be worth exploring in more robust studies.

The major strength of this study is that includes five years of data with nearly 400,000 ED attendances. Limitations include this being a single centre study and the results may have limited generalisability. However, our results are similar to those of previously published research and our findings may be applicable in other academic type 1 emergency departments. Second, this was an analysis of routinely collected data and we did not have access to several important variables that may impact upon breach probability including specific information on disease severity, sex, triage score, discharge disposition, staff training levels (junior vs non junior physicians), social and economic deprivation, and direct measures of department congestion such as bed occupancy, match of supply and demand (e.g., overall ED census to staffing), door to provider time, and inpatient flow (e.g., boarding).

## Conclusions

In summary, we have identified a number of factors associated with the probability of breaching the four-hour emergency department wait time target including patient age, ED referral source, the types of investigations patients receive, as well as the hour, day, and month of arrival to the ED. Patients most likely to breach the four-hour target were older, presented at night, presented on Monday, received multiple types of investigation in the ED, and were not self-referred. The results of this study may be used by policy- and decision-makers to identify possible approaches to reducing breaching of the four-hour performance target, including overnight staffing on select weekdays, expertise of clinicians undertaking assessments, expanding access to imaging and diagnostic tests, and rapid discharge facilities.

## Abbreviations

A&E: accident and emergency
CI: confidence interval
ED: emergency department
NHS: national health service
OR: odds ratio
